# Spatiotemporal dynamics of re-innervation and hyperinnervation patterns by uninjured CGRP fibers in the rat foot sole epidermis after nerve injury

**DOI:** 10.1186/1744-8069-8-61

**Published:** 2012-08-30

**Authors:** Liron S Duraku, Mehdi Hossaini, Sieske Hoendervangers, Lukas L Falke, Shoista Kambiz, Vivek C Mudera, Joan C Holstege, Erik T Walbeehm, Tom J H Ruigrok

**Affiliations:** 1 Department of Neuroscience, Erasmus MC, University Medical Center, Rotterdam, The Netherlands; 2 Department of Plastic, Reconstructive and Hand Surgery, Erasmus MC, University Medical Center, PO Box 2040, 3000 CA, Rotterdam, The Netherlands; 3 The Institute of Orthopedics and Musculoskeletal Sciences, Royal National Orthopedic Hospital, University College London, Stanmore, UK

**Keywords:** SNI, PGP 9.5, CGRP, Substance P, NF-200, P2X3, Epidermis

## Abstract

The epidermis is innervated by fine nerve endings that are important in mediating nociceptive stimuli. However, their precise role in neuropathic pain is still controversial. Here, we have studied the role of epidermal peptidergic nociceptive fibers that are located adjacent to injured fibers in a rat model of neuropathic pain. Using the Spared Nerve Injury (SNI) model, which involves complete transections of the tibial and common peroneal nerve while sparing the sural and saphenous branches, mechanical hypersensitivity was induced of the uninjured lateral (sural) and medial (saphenous) area of the foot sole. At different time points, a complete foot sole biopsy was taken from the injured paw and processed for Calcitonin Gene-Related Peptide (CGRP) immunohistochemistry. Subsequently, a novel 2D-reconstruction model depicting the density of CGRP fibers was made to evaluate the course of denervation and re-innervation by uninjured CGRP fibers. The results show an increased density of uninjured CGRP-IR epidermal fibers on the lateral and medial side after a SNI procedure at 5 and 10 weeks. Furthermore, although in control animals the density of epidermal CGRP-IR fibers in the footpads was lower compared to the surrounding skin of the foot, 10 weeks after the SNI procedure, the initially denervated footpads displayed a hyper-innervation. These data support the idea that uninjured fibers may play a considerable role in development and maintenance of neuropathic pain and that it is important to take larger biopsies to test the relationship between innervation of injured and uninjured nerve areas.

## Background

Noxious stimuli in the epidermis are detected by free nerve endings of thinly myelinated (Aδ) or unmyelinated (C) fibers, which are termed nociceptors [1,2]. Damage to nociceptive fibers often leads to neuropathic pain [3], however, its etiology is only poorly understood. Epidermal nociceptive fibers can also be subdivided into peptidergic fibers that contain neuropeptides like calcitonin gene-related peptide (CGRP) and/or substance P [4], and non-peptidergic fibers that are identified by isolectine B4 (IB4) [5]. It is specifically the group of peptidergic fibers that is suspected to play an important role in the induction and maintenance of neuropathic pain [6].

Several animal models of neuropathic pain have been used to study the anatomical changes of epidermal fibers after peripheral nerve injury [7-9]. After a chronic constructive injury (CCI) of the sciatic nerve, a sustained decrease in CGRP epidermal fibers was found together with the development of neuropathic pain behavior [7,10]. However, using the same model, neuropathic pain has also been reported in combination with an increase of upper-dermal CGRP fibers [8,11]. Paradoxical results showing both increased and decreased densities of epidermal fibers were also reported for the affected skin of patients suffering neuropathic pain [12,13]. As yet, these differences have not been related to specific types of nerve injury. The CCI model, which uses catgut ligatures around the sciatic nerve, often results in a local inflammatory reaction, which commonly leads to a local, but variable, compression injury rather than a transection [14-16]. Therefore, it is unclear to what extent changes in density of fibers are due to re-innervation by injured nerves or by collateral sprouting of uninjured nerves from adjacent areas.

**Figure 1:**
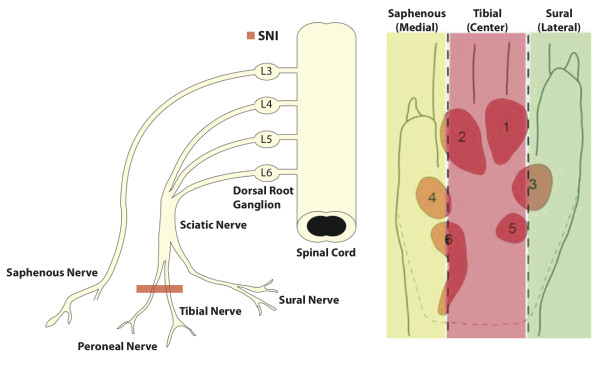
**The Spared Nerve Injury (SNI) procedure consists of transection and displacement of the tibial and common peroneal nerves, sparing the adjacent sural and saphenous nerves.** This procedure leads to complete denervation of the tibial (central) innervated area (red), but leaves the medial (saphenous: yellow) and lateral (sural: green) sides of the hind paw glabrous skin intact.

In our present study we have determined the effect of nerve transection on subsequent changes in pain thresholds of the different foot sole areas and co-related this to changes in the density of epidermal CGRP fibers in a rat model. For this purpose we have used the spared nerve injury (SNI) model for neuropathic pain in which the common peroneal and tibial nerves are transected leaving the saphenous and sural nerves intact (Figure 1) [17]. This procedure denervates the central area of the glabrous skin of the hind paw, but not its medial- and lateral-most areas, and results in the development of neuropathic pain as indicated by hypersensitivity of the intact areas. This procedure better resembles clinical situations where peripheral nerves are transected by trauma or surgery. Moreover, it results in a well-defined separation between injured (peroneal and tibial nerve) and uninjured (saphenous and sural nerves) nerve fibers. 

## Results

### Mechanical withdrawal threshold

**Figure 2:**
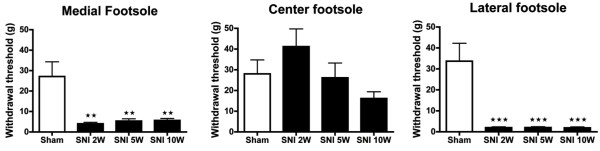
**Histograms showing the mechanical threshold (± SEM) in the lateral, center and medial territories of the affected hind paw glabrous skin as determined with the Von Frey test.** The SNI group showed a significantly decreased mechanical withdrawal threshold in the medial and lateral foot sole at 2, 5 and 10 weeks PO compared to the Sham group. Center area of the foot sole of the SNI group showed a somewhat higher mechanical withdrawal threshold, which decreased in week 10 compared to the Sham group, which were not significant. N = 6 per group. One-way ANOVA with post-hoc Turkey test, unpaired t-test *: P < 0.05, **: P < 0.01, ***:P < 0.001.

Two weeks after the SNI procedure, a severe decrease in the mechanical withdrawal threshold, as determined with the Von Frey test was noted at both the medial and lateral side of the affected foot sole (Figure 2). This implied a significantly increased sensitivity of these parts, which was seen to persist at 5 and 10 weeks postoperatively (PO). The mechanical withdrawal threshold on the lateral side of the foot sole (sural territory) was somewhat lower than on the medial side (saphenous territory) of the affected hind paw at 2, 5 and 10 weeks post-surgery, however this did not reach statistical significance. Testing the center area of the foot sole showed a higher mechanical threshold (implying reduced sensitivity) 2 weeks PO, however not significant, that reversed after 5 weeks and 10 weeks PO as compared to sham-treated levels. There was no significant change in the mechanical withdrawal threshold of the sham-operated rats.

### Hot and cold withdrawal threshold

**Figure 3:**
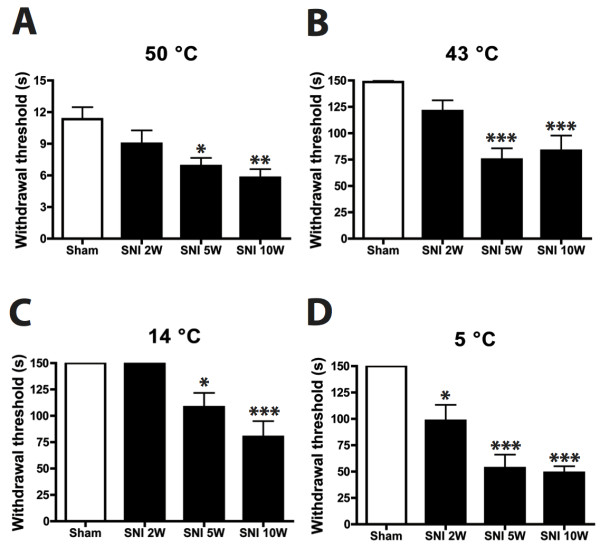
**Histograms showing the temperature withdrawal thresholds of the affected hindpaw glabrous skin as determined by the hot and cold plate test.** The SNI group shows a significantly lower paw withdrawal threshold at 50 and 43°C after 5 and 10 weeks postoperatively as compared to the sham group. Also at 14 and 5°C the SNI group has a significantly lower withdrawal threshold as compared to the Sham group only at 5 and 10 weeks. N = 9 per group. One-way ANOVA with post-hoc Turkey test, unpaired t-test *: P < 0.05, **: P < 0.01, ***:P < 0.001.

Temperature sensitivity was tested by placing the animals on a temperature-controlled surface using a warm and a hot temperature (43°C and 50°C) and two graded cold (14°C and 5°C) temperatures (Figure 3). At 2 weeks PO the SNI rats showed a non-significant tendency of having a lower paw withdrawal threshold of the affected hind paw as compared to the sham group for the hot (50°C) surface. This became significant at 5 and 10 weeks PO for both tested temperatures (Figure 3A,B) and imply an increased sensitivity for high temperatures of the SNI group as compared to the sham group. Essentially similar but inverse results were obtained for the cold plate test of 14°C and 5°C (Figure 3C,D). Note, however that the SNI group already demonstrated a significantly lower withdrawal threshold at 2 weeks PO.

The results of the behavioral tests show that the SNI procedure results in both a prominently increased mechanical and temperature sensitivity reflecting hyperalgesia/allodynia and subsequently suggest that these animals suffer neuropathic pain.

### The distribution of epidermal CGRP-IR fibers in foot sole in the control group

**Figure 4:**
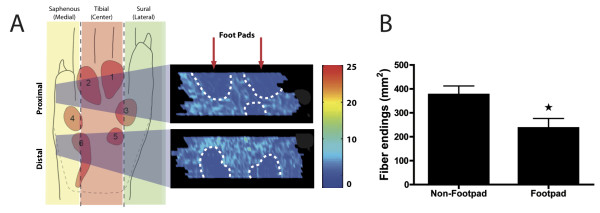
**Epidermal CGRP-IR 2-D density profile of the hind paw glabrous skin.A**: Two large skin biopsies (left hand panel, grey shaded areas) are taken from the distal and proximal foot sole and cut transversely. Serial sections were plotted, binned and the density of labeled profiles was visualized in a 2-D density profile that was made using Matlab routines (colorbar reflect number of labeled fibers.) The resulting plots indicate that the footpads are less densely innervated compared to non-footpad areas. **B**: Quantification shows a significant lower number of epidermal CGRP-IR fibers in the footpads compared to non-footpads. (N = 5), student t-test, *P < 0.05.

The labeling patterns of CGRP fibers in the skin of the foot sole of the sham-operated (control) group were consistent with results reported in previous studies [4]. In control skin, we found that CGRP-IR fibers form thick fiber fascicles in the superficial and deeper dermis. Most of these CGRP-IR fiber fascicles penetrate the epidermis from which individual fibers leave the fascicle, taking a usually orthogonal course to the surface while displaying several varicosities. Using the serial Neurolucida reconstructions (based on 1 out of every 8 sequential sections) a two-dimensional density profile of terminal CGRP-IR fibers was constructed. This profile showed that the density of epidermal CGRP fiber endings in the foot sole was significantly (p < 0,001) lower in the footpads as compared to surrounding non-footpad regions (Figure 4). 

### Spatiotemporal changes in the distribution of epidermal CGRP-IR fibers in the SNI model

**Figure 5:**
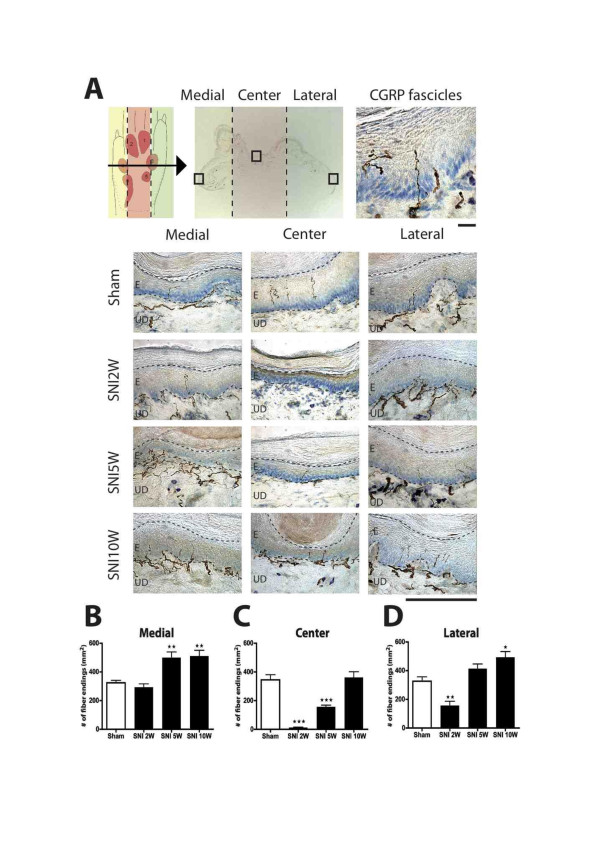
**Micrographs showing staining for CGRP-IR fibers in skin biopsies taken from the medial (saphenous), center (tibial) and lateral (sural) part of the foot sole (Figure**5**A).** Note that two weeks after SNI a complete loss of CGRP-IR fibers is observed in the central part while preserving CGRP-IR fibers on the medial and lateral side. Five weeks PO, the SNI group shows an increase of CGRP-IR fibers in the medial and lateral part when compared to the sham group. Also, at this time a re-innervation of the center area is noted. Ten weeks PO, the SNI group shows CGRP-IR fibers in all three parts of the foot sole, suggesting complete re-innervation of CGRP-IR fibers in the foot sole. Upper scale bar: 25 μm, and lower scale bar: 250 μm. Diagrams showing CGRP-IR fiber endings (mm^2^) in the medial, center and lateral area of the hind paw glabrous skin. In the medial area there is a significantly higher density of epidermal CGRP-IR endings at 5 and 10 weeks PO (**Figure **5B-D). The center area shows a decreased number of CGRP-IR fibers at 2 weeks, which normalizes at sham levels at week 10. Lateral area shows an initial decrease of CGRP-IR fibers at 2 weeks that becomes significantly higher at 10 weeks PO. (n = 6) One-way ANOVA with post Turkey test *: P < 0.05, **:P < 0.01, ***:P < 0.001.

Figure 5 depicts microphotographs of transverse sections at the level of footpads 3 and 4, showing the appearance of CGRP-IR fibers in the medial, center and lateral areas of the foot sole of sham- and SNI-treated rats after at 2, 5 and 10 weeks PO. From these graphs, it is evident that, two weeks after SNI the center region was completely devoid of CGRP-IR fibers. However, at 5 weeks PO, and especially at 10 weeks PO, a re-innervation of CGRP-IR fibers in this region was noted. From the microphotographs no obvious differences could be discerned of changes in the density in the areas (i.e. medial and lateral) that were not innervated by the transected nerves. Using quantitative data generated on the density of CGRP-IR fibers at the medial, center and lateral region of the foot sole (see Methods), both the density of the number of CGRP-IR fibers that crossed the border between the dermis and epidermis as well as the number of terminal CGRP-IR fibers that seemed to terminated within the epidermis were determined. As analysis of both sets of data revealed similar changes, we have only described the results of the latter quantification (i.e. based on terminal epidermal fibers, however also see Figure 9 described below).

**Figure 6:**
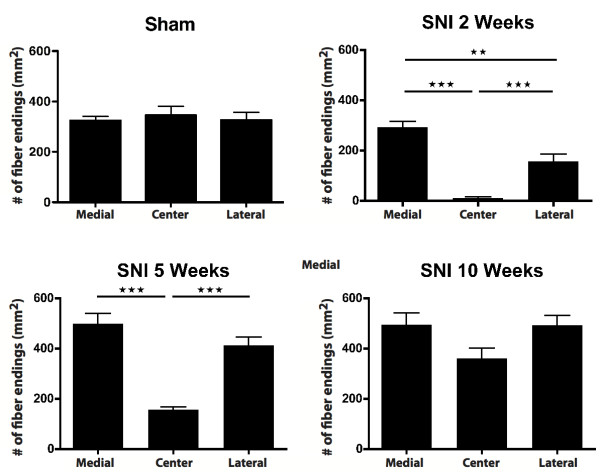
**Diagrams showing the number of epidermal CGRP-IR fibers (mm**^**2**^**) in a group.** The lateral area has a significantly lower number of CGRP-IR fibers compared to the medial area at 2 weeks PO, which normalizes at week 5. At 10 weeks PO there is no significant difference between the medial, center and lateral area of the hind paw glabrous skin of the SNI treated animals. (n = 6) One-way ANOVA with post Turkey test *: P < 0.05, **:P < 0.01, ***:P < 0.001.

The overall density of epidermal CGRP fibers in the foot sole (as indicated by the average number of terminal fibers per mm^2^) was significantly lower for the SNI group at 2 and 5 weeks PO as compared to the control group, but these returned to control values at 10 weeks PO (data not shown). The density of CGRP-IR fibers of two additional SNI rats with a survival period of 4 months showed no significant difference in epidermal CGRP-IR fibers density with the 10-week SNI group (data not shown). Therefore, 10 weeks was considered to be a stable final state for regeneration of CGRP fibers in this model. Figures 5 and 6 show a temporal and spatial comparison, respectively, of the changes in the density of CGRP-IR epidermal fibers. Indeed, these results demonstrate quite clearly that at two weeks PO, the center region is almost completely devoid of CGRP-IR fibers (Figure 5C, Figure 6). At this time point, the innervation of the medial region of the foot sole of SNI-treated rats still resembles that of sham-operated rats (Figure 5B), whereas the lateral side shows an unexpected but clearly reduced density of CGRP-IR fibers (Figure 5D, Figure 6). Five and 10 weeks after SNI, a gradual re-innervation of epidermal CGRP-IR fibers in the center region was found having a density similar to that of sham-operated animals at 10 weeks PO (Figure 5C). Remarkably, this coincided with an increase (with respect to the sham group) in density of CGRP-IR epidermal fibers of the medial and lateral parts of the foot sole. This was first noted at the medial side (at 5 weeks PO) but later also at the lateral side of the foot sole (Figure 5B, D). Note that although the density of CGRP-IR fibers at the lateral side of the foot was not significantly different from that of sham animals at 5 weeks PO, it was considerably increased with respect to the situation at 2 weeks PO (Figure 5D).

**Figure 7:**
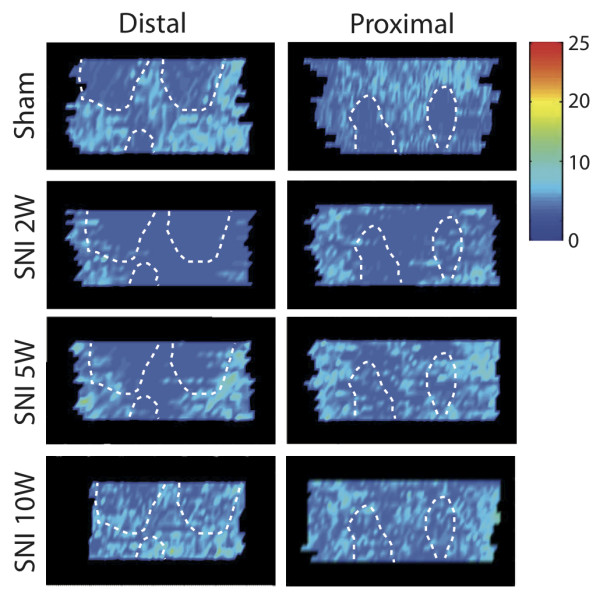
**Two-dimensional density profile of epidermal CGRP-IR fibers in the distal and proximal parts of the foot sole.** Note patches with lower CGRP-IR density in the sham group, which represent the footpads. After 2 weeks PO there is a complete abolishment of CGRP-IR fibers in the center area (tibial). Five weeks PO an enhanced density of epidermal CGRP-IR fibers within the medial and lateral areas can be observed. Ten weeks PO the epidermal CGRP-IR fiber density profile shows re-innervation of fibers into the previously denervated center area (tibial). Note that the lower density in the footpads that is seen in the sham group is not observed at 10 weeks PO in the SNI group.

**Figure 8:**
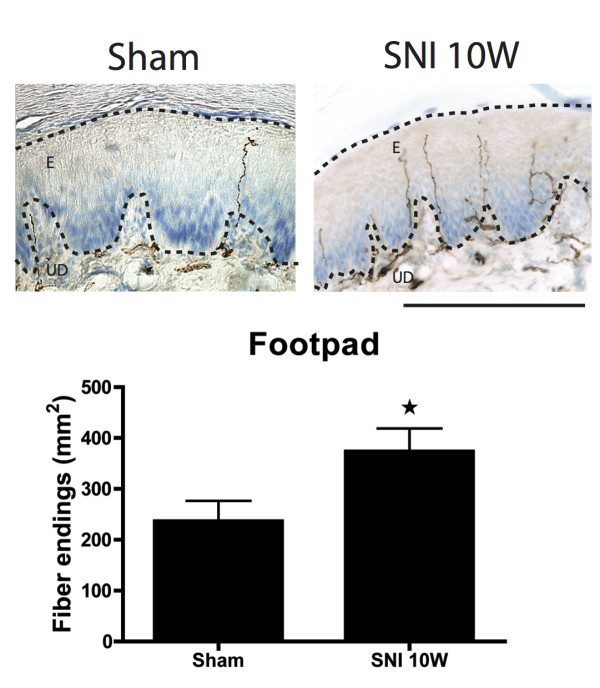
**Micrographs showing footpad epidermal CGRP-IR fibers in the Sham versus SNI 10 week groups.** There are a significant higher number of epidermal CGRP-IR fibers in the footpads of the SNI group compared to the Sham group. (n = 5), student t-test, * :P < 0.05. Scale bar: 250 μm.

Two-dimensional density profiles of CGRP-IR terminal fibers in the epidermis of the foot sole were prepared of selected specimens at all PO time points and compared to density plots of sham-operated rats. We have already described that normally the footpads display a conspicuously lower density of CGRP-IR epidermal fibers with respect to the non-footpad areas of the foot sole (Figure 4, Figure 7). The density profiles also show that two weeks PO a clear decrease of epidermal CGRP-IR fibers in the center area of the foot sole can be seen, which becomes re-innervated from the medial and lateral areas of the glabrous foot sole at week 5. Interestingly, the footpad areas that are normally innervated with relatively low numbers of epidermal CGRP-IR fibers could no longer be distinguished in the SNI rats at 10 weeks PO (Figure 7). This indicates that the density of the CGRP-IR fibers in the epidermis of the footpad was considerably increased compared to normal. Quantification of the number of CGRP-IR fibers in a pre-determined part of the footpad of Sham and SNI animals at 10 weeks PO further confirmed this finding (Figure 8).

### Intra-epidermal sprouting

**Figure 9:**
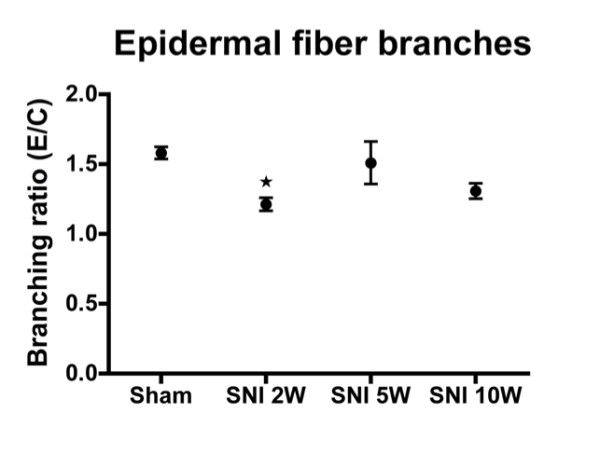
**Histogram showing branching ratio of epidermal CGRP-IR fibers as determined by the number of epidermal endings (E) divided by the number of fibers crossing the dermis/epidermis boundary (C).** Note that after an initial decrease of branching ratio these levels are quickly normalized. (n = 6), One-way ANOVA with post Turkey test, *: P < 0.05.

Changes in the density of the epidermal terminal CGRP-IR fibers can also occur due to increased sprouting of re-innervating fibers that enter the epidermis. In order to test this the number of epidermal endings in the medial, center and lateral region of the foot sole were divided by the number of fibers that crossed the dermal-epidermal border. This sprouting ratio at 2 weeks PO was somewhat decreased in all regions (medial, center and lateral), but was not significantly different at the other examined PO time points (Figure 9). We conclude that increases in the density of epidermal CGRP-IR endings cannot be attributed to changes in the epidermal sprouting ratio.

### Epidermal thickness of the foot sole areas

**Figure 10:**
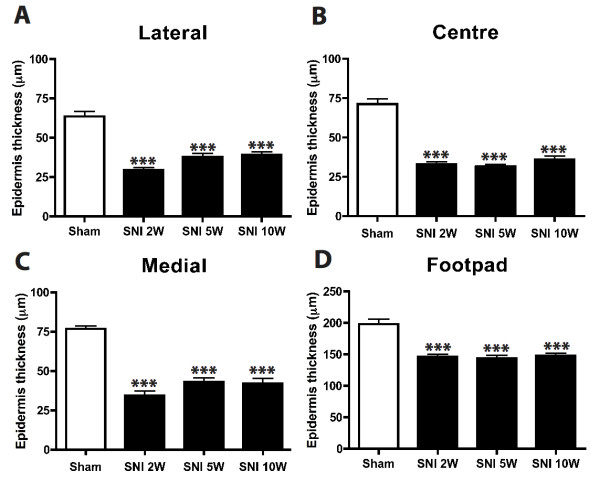
**Shows the epidermal thickness of the lateral, centre and medial part of the ipsilateral foot sole.** It is evident that all the regions in the ipsilateral side have a significant thinner (p < 0,001) epidermis compared to the sham- treated group. (N = 6), One-way ANOVA with post Turkey test ***:p < 0,001.

Previous reports showed that a crush injury to the sciatic nerve resulted in a significant reduction in epidermal thickness that was accompanied with a substantial decrease in epidermal nerve fibers [18-20]. Because this reduction in epidermal thickness was reversed when fibers re-innervate the denervated epidermis, it was concluded that there is a positive correlation between the epidermal fiber density and epidermal thickness after peripheral nerve injury. In order to see if the observed changes in density of CGRP-IR fibers in the SNI model also correlate with the thickness of the epidermis we have quantified the epidermal thickness of the lateral, center and medial foot sole. In general, the epidermal thickness in the SNI group (n = 6) was significantly reduced with respect to the sham treated animals (n = 6, p < 0.001) at all PO time points (Figure 10). Hence, a relation between the hyper-innervation of the lateral and medial part of the foot sole and re-innervation of the center part and the thickness of the epidermis could not be established. 

### Evans blue staining

To exclude that there is any re-innervation from the transected tibial and peroneal nerve in the SNI treated animals we performed Evans blue staining of the innervation areas of the tibial and peroneal nerve in the sham (N = 4) and SNI-treated group (N = 4) at 10 weeks PO. As expected, for sham-operated animals, stimulation of the tibial nerve resulted in blue coloring of the plantar surface of the foot. It is interesting to note that the footpads do not show the same intense blue coloring as the non-footpad area, which is in accordance with the decreased epidermal CGRP-IR density of the footpads. Stimulation of the peroneal nerve elicits blue coloring of the dorsal aspect of the foot. Both innervation areas are in accordance with previous reports [17]. In contrast, after stimulating the tibial and peroneal nerve in a SNI-treated rat, there is no Evans blue colouring, implying indirectly that the re-innervation with CGRP fibers in the center area has to come from the uninjured medial (saphenous) and lateral (suralis) areas.

## Discussion

In this study the following key observations were made: 1) The distribution of CGRP-IR fibers in the epidermis of the footpads is lower compared to that of the glabrous skin of the foot sole. 2) Denervation of the central foot sole area by the SNI procedure results in a prominent and persistent mechanical hypersensitivity of the lateral and medial sides of the foot sole and in heat and cold hyperalgesia and allodynia. 3) The central area of the foot sole including the footpads re-innervates with CGRP-IR fibers from the lateral and medial sides.

### CGRP innervation of the normal rat foot sole

Most studies on the innervation of the foot sole use relatively small sample areas and do not distinguish between innervation of footpad and non-footpad areas [21-23]. Plantar footpads on the rat foot sole consist of six cushion-like structures [4,7,10]. Most of the foot sole area was examined in this study and a lower density of CGRP-IR epidermal fibers in the footpads compared to the surrounding regions was observed. The EB experiment showing poor footpad coloring confirmed this finding. This may have implications on how rodents perceive tactile stimuli and how sensation of the foot should be tested. Von Frey testing for mechanical sensitivity typically uses the center or lateral margins of the foot, usually avoiding the footpads. In thermal tests, however, the footpads are more likely to contact the test surface. Therefore, in experiments assessing mechanical versus thermal response properties in rats, the innervation differences of footpad and non-footpad areas should be taken into account. In the current study we have found that heat (50º and 43°C) and cold hyperalgesia (5°C) and cold allodynia (14°C) reach significant levels at 5 and 10 weeks PO. The hyper-innervation of CGRP-IR fibers in the footpads of the SNI-treated animals may account for the thermal hyperalgesia and allodynia, because of the direct contact with the thermal plate.

### Neuropathic pain and density of CGRP innervation of the epidermis

Our study shows an almost complete loss of CGRP-IR fibers in the center area, following an SNI procedure, whereas the density of the medial part of the foot sole remains unchanged until 5 weeks PO when an increase in density was noted. At the lateral side of the foot, the density of CGRP-IR fibers initially decreased (at 2 weeks PO) but recovered at 5 weeks PO and increased at 10 weeks. Nevertheless, by 2 weeks PO mechanical hypersensitivity at both edges of the foot sole was noted. Because electrophysiological changes accompanying neuropathic pain behavior have also been reported to occur in uninjured fibers as early as one week PO in partial peripheral nerve injury models [24,25], it seems likely that electrophysiological and behavioral changes precede detectable changes in morphology. Interestingly, it has also been shown that after a ventral rhizotomy, which results in degeneration of myelinated fibers that in turn leads to neuropathic pain behavior in the rat. The postulated mechanism is that through Wallerian degeneration reacting Schwann cells produces factors that diffuse to nearby uninjured unmyelinated C-fibers and alter their properties [26]. In addition, degeneration of myelinated motor fibers leads to mitogenic changes to nonmyelinating uninjured Schwann cells with intact axons, which in turn may play a significant role in the development of neuropathic pain [27,28]. However, it is also possible that other subtypes of sensory fibers may show morphological changes that correlate more precisely with the mechanical hypersensitivity. However, preliminary data from our group shows that the peptidergic Substance P, non-peptidergic P2X3 and myelinated NF-200 fibers do not show hyper-innervation of the epidermis in SNI-rats as compared to sham. More specifically the non-peptidergic P2X3 fibers exhibit almost no re-innervation as described in an earlier study [8]. However to discuss this in more detail is out of the scope of the current study.

The sural nerve-innervated lateral aspect of the foot sole has a more severely increased mechanical pain threshold compared to the medial side (innervated by the saphenous nerve). Although agreeing with previous results [17], this seems at odds with the observed initial lateral reduction in density of CGRP-IR fibers. The latter effect indicates that there may be considerable spatial overlap in innervation of the tibial and sural nerves. We suggest that the observed medio-lateral differences in sensitivity at 2 weeks PO could be related to the fact that the saphenous nerve is mostly derived from the L3 dorsal root ganglion where there is less interaction between injured and non-injured neuronal cell bodies (Figure 10) as compared to the L4-L6 ganglia from which the sural nerve is derived [29]. This may lead to a delayed increase of CGRP-IR epidermal fibers on the sural side compared to the saphenous side, while the sural side has a lower mechanical threshold.

Although the SNI procedure leads to a virtually complete absence of CGRP-IR epidermal fibers in the center area of the foot sole, only a marginally increased mechanical withdrawal threshold nor a complete absence of the withdrawal reflex were noted. This could be due to stimulation of the hypersensitive lateral or medial region of the foot sole, which is inevitable when applying forces exceeding 40 g. At 5 and 10 weeks PO the observed re-innervation of epidermal CGRP-IR in the center area is in accordance with a return of the mechanical withdrawal threshold to control levels.

The increase in epidermal CGRP-IR fibers might be an increase in expression levels of CGRP and not an increase of fibers. It is known that after peripheral nerve injury the uninjured DRG neurons, adjacent to injured neurons, increase CGRP expression through the p38 mitogen activated protein kinase (MAPK) pathway [30,31], consequently leading to increased peripheral transport and filling of nerve terminals in already existing peripheral but undetected in control conditions. However, the increased density of CGRP-IR fibers in the sural (lateral) and saphenous (medial) territories found at 5 weeks PO, is in accordance with a previous study where, in the SNI model, an increased density was observed in small biopsies of the lateral hind paw area using PGP 9.5, which is a marker for all types of nerve fibers [9]. Therefore the collateral sprouting of CGRP-IR fibers in the SNI model seems more plausible. Other studies, using the CCI neuropathic model, which also leads to thermal and mechanical hypersensitivity [32], have reported both normal as well as inverse correlations between neuropathic pain and density of nociceptive fibers [7,8,10,11]. From these studies it appears that the occurrence and severity of neuropathy in CCI models is highly variable [33-37], which may relate to the varying results in re-innervation patterns in hind paw glabrous skin. The variability in CCI results is likely to be related to inconsistent tightness of the ligatures, which may result in a variable percentage of permanently damaged axons [29]. The CCI procedure also has an inflammatory component, evoking intraneural edema, also resulting in variable constriction [14,15]. Therefore, it is uncertain if the changes in skin innervation are due to injured, uninjured or a combination of these two groups of fibers.

In the SNI model, the injury consists of a complete transection of nerve fibers, which are subsequently prevented from regrowing by the ligation as was substantiated by the lack of Evans Blue extravasation at 10 weeks PO in SNI rats. Therefore, the re-innervation patterns of epidermal CGRP-IR fibers in the SNI model must originate from uninjured fibers located in adjacent areas. As such, we propose that the SNI model has distinct advantages over the CCI model for studying re-innervation of denervated skin by undamaged axons.

### Effect of SNI on epidermal thickness

Our results show that, in contrast to previous reports [18,19,38], there is an inverse correlation between epidermal thickness and density of intra-epidermal fibers. Peptidergic epidermal fibers have been suggested to promote proliferation of keratinocytes and maintenance of skin tropism [39,40]. It should be pointed out, however, that the rats in these studies did not suffer chronic neuropathic pain as is the case in the SNI model. Keratinocytes not only produce CGRP-β (whereas fibers mostly contain CGRP-α), likely to have an autocrine/paracrine role in epidermal homeostasis [41], but also release β-endorphin, which, by binding to μ-opioid receptors located on CGRP containing sensory endings, seems to ameliorate heat-pain induced withdrawal [42]. Although CGRP-β is normally expressed at low levels and rather heterogeneously among epidermal keratinocytes, chronic pain conditions induces an increased and more homogeneous expression [41]. Therefore, we suggest that the combination of chronic pain, increased densities of CGRP-IR fibers and potentially also of increased CGRP-β release by non-proliferating keratinocytes are instrumental in preventing epidermal thickening.

## Conclusion

The mechanism of sprouting of intact fibers is still poorly understood. Sprouting of epidermal CGRP-IR fibers is likely to be triggered by enhanced levels of Nerve Growth Factor (NGF), which is secreted by Schwann cells and other non-neuronal cells (e.g. epidermal cells) after nerve lesion [8]. In the CCI model, intact nerve fibers are surrounded by high quantities of NGF binding on the high affinity receptor tyrosine kinase A receptor (TrkA) [11], which in turn may induce sprouting of both these fibers. In the SNI model the sprouting ratio suggests that the formation of new branches mostly occurs in the dermis. Because NGF is also produced by the epidermal cells (e.g. keratinocytes) and plays a role in the proliferation and maintenance of the epidermis [43], we hypothesize that peripheral nerve injury increases the level of NGF significantly in the denervated epidermis. This not only affects the epidermal cells but also diffuses into the upper dermal area spreading in lateral directions and subsequently inducing sprouting of CGRP-IR fibers. These then enter both the normal as well as the denervated epidermis. The hyperinnervation of normal skin and the as yet unexplained enhanced invasion of CGRP-IR fibers into the footpads may also be related to the development of neuropathy.

## Methods

### Animals

Experiments were performed on adult male Wistar rats (n = 42) (includes 2 rats of 4 months post-surgery SNI treatment). All experiments were approved by the Dutch Ethical Committee on Animal Welfare (DEC) and all procedures adhered to the European guidelines for the care and use of laboratory animals (Council Directive 86/6009/EEC).

### The spared nerve injury (SNI) model

For this experiment we used the SNI procedure described by Woolf and Decosterd (2000). Under isoflurane (2%) anesthesia the skin on the left lateral surface of the thigh was incised and the biceps femoris muscle was divided and spread lengthwise to expose the three branches of the sciatic nerve: i.e. the sural, common peroneal and tibial nerves. The tibial and common peroneal nerves were ligated together with 5–0 silk suture and cut approximately 2 mm distal to the ligation (Figure 10). Great care was taken to avoid any contact with or stretching of the intact sural nerve. This procedure denervates the central part of the footsole while leaving the innervation of both the lateral and medial side of the footsole intact [17]. Sham controls involved only the exposure of the sciatic nerve and its branches without any subsequent handling or lesioning. The skin was sutured and the animals were allowed to recover. In all cases, postoperative analgesia was provided by subcutaneous administration of buprenorphine (0.05-0.1 mg/kg; Temgesic; Schering-Plough BV, Utrecht, the Netherlands). Animals were monitored daily for signs of stress or discomfort but in all cases recovered uneventfully with no autotomy.

### Behavioral experiments

The development and level of neuropathic pain was determined in the affected hind limb 2, 5 and 10 weeks after the SNI procedure. As a control, sham operated animals were tested at the same time points. Two tests were used to examine neuropathic pain development.

#### Von Frey test

At 2, 5 and 10 weeks postoperatively (PO), the mechanical threshold of the hind paws was measured using Von Frey hairs ranging from 0.6 to 300 g in a set of 14 filament steps [44]. In the current experiment we used up-down testing starting with low force followed by gradually increasing forces. Rats were placed in a plastic box with a mesh floor, in which the rats were able to move freely. Each Von Frey hair was applied for 2 s (1 trial) at 5 s intervals; the threshold for a positive test was set at 3 trials, which evoked responses out of a maximum of 5 trials. A response consists of a flinch or flutter of the tested limb. The medial and lateral sides were stimulated at the transition point from glabrous to hairy skin at either side of footpad 3 and 4 (Figure 10). The center foot sole was stimulated at its very center between footpad 3 and 4.

#### Hot/Cold plate test

The hot/cold plate test [45,46] consists of one aluminum plate (21 × 21 cm) with a see-through Plexiglass cage, in which channels are placed in a spiral form to provide a homogenous adjustment of the plate temperatures. These channels are filled with water that is regulated by a water bath (HAAKE K20), with a temperature range from 0 to 50°C, a maximum pressure of 300 mbar and a maximum flow rate of 12.5 L/min. The water bath is connected with PVC tubes to the aluminum plates and pumps the water through the plates. We measured the plate temperatures with thermocouples (J-type: Fe/Cu-Ni) at the center of each plate. The plate temperatures corresponded with the water bath temperatures with a maximum discrepancy of ± 0.5°C. The rats were placed on the hot or cold plate, and the paw withdrawal latency was measured. The measurement started from the first contact of the hind paw with the plate and the time until a positive response was the latency time. Paws lifts are defined as a quick flutter or flinch of the affected hind paw.

### Tissue preparation

Following survival periods of 2, 5 and 10 weeks, all animals received an overdose of pentobarbital (100 mg/kg). Two additional rats were examined after a survival of 4 months. The sham animals were sacrificed at 2 weeks post-surgery and compared to two naïve animals in thermal and mechanical behavior as well as the number of epidermal CGRP fibers in the foot sole (data not shown). There was no significant difference between sham animals post-surgery 2 weeks and naïve rats. Therefore we took 2 weeks post-surgery for the sham animals as a safe time-point to compare to the SNI-treated rats. Under deep anaesthesia the glabrous skin of the affected hind limb was dissected in a distal and proximal strip of tissue, which were immersion-fixed in 2% paraformaldehyde-lysine-periodate for 24 hours at 4°C. The skins were embedded together with the extracted rat brain in a gel containing 10% gelatin, 4% formaldehyde and 30% sucrose [47]. Thereafter, transverse sections were cut at 40 μm and collected serially in glycerol in eight numbered vials for long-term storage at −20°C. Using the brain as an anatomical marker in the gelatin-embedded skin tissue enabled serially mounting of the free floating sections of each vial.

### Immunohistochemistry

The sections were pre-incubated (90 min, room temperature (RT)) in a mixture containing bovine serum albumin (BSA 2%) phosphate buffered saline (PBS, pH 7,4). (Fraction V, Roche) and 0.5% Triton X-100. Thereafter, the sections were rinsed in PBS and incubated for 48 hours in a cocktail of 2% BSA containing goat anti-CGRP antibody (Calbiochem, PC205L) (1/30.000). Subsequently, sections where incubated with a secondary antibody rabbit anti-goat for 90 min room temperature. Sections were further processed using a Vectastain Elite ABC kit (Vector, Burlingame, CA) (90 min at RT). Finally, 3,-3′ diaminobenzidine (DAB) enhanced by the glucose oxidase-nickel-DAB method (Kuhlmann and Peschke, 1986) was used to identify antigenic sites. The sections were mounted on gelatinized slides, air dried overnight, counterstained with thionine, dehydrated using absolute ethanol (< 0.01% methanol), transferred to xylene, and coverslip mounted with Permount (Fisher, Hampton, NH). The CGRP antibody used in the present experiments has been extensively characterized earlier [48].

### Evans Blue experiment

The effect of the SNI lesion on the innervation areas of the hind paw skin was also tested by visualizing the extravasation response using Evans Blue (EB) and electrical stimulation of the peroneal and tibial nerves [49]. This extravasation response was determined in four SNI (two tibial and two peroneal nerve stimulations) at 10 weeks PO and in four sham-operated rats (two tibial and two peroneal nerve stimulations). In order to facilitate i.v. injection of EB, the animals were placed on a 38°C surface to induce vasodilatation. Subsequently, the animals were anesthetized with 3% isoflurane and both paws were depilated using depilatory-crème ‘Veet’ to acquire a clear image of the skin. The sciatic nerve and its tibial and peroneal branches were exposed and carefully inserted in a silicone cuff (diam. 3 mm) that could be opened length-wise and in which anodal and cathodal electrodes were embedded. Proximal to the stimulation site a crush injury was applied to prevent central propagation of the stimulation [50]. This procedure took place under microscopic guidance (Zeiss OP-MI 6-SD; Carl Zeiss, Goettingen, Germany) to minimize damage to the distal part of the nerve. A solution of Evans blue (2% EB solved in 0.9% saline, 4 ml/kg body mass) was injected in the tail vein of the rat. Stimulation of the nerve was started 5 minutes after EB injection and lasted for a period of 10 minutes (10 Hz, 0.5 ms, 12 mA: Viking stimulator, Nicolet Biomedical IES405-2). The skin area of the foot innervated by the stimulated nerve exhibited the characteristic deep blue coloration indicating extravasation of Evans blue. Ten minutes after stimulation, the animals were sacrificed by an overdose of pentobarbital (100 mg/kg). Additional extravasation does not occur 10 minutes after stimulation [51]. The animals were not handled during this period to exclude artifactual blue coloration by mechanical stimuli. The affected hind limb was amputated at the level of the ankle and fixed in ethanol. Three-dimensional images (Cys3 fluorescence, 545 nm/30 nm exciter, 610/75 nm emitter) were obtained using a Bioptronics 3D Medical Imaging system.

### Analysis of CGRP terminal fibers

Every 8^th^ section of both the distal and proximal strips of the foot sole was used for analysis. These sections were arranged in serial order from proximal to distal using reference brainstem sections that were embedded together with the skin tissue [47]. The sections were analyzed by systematically identifying the location of CGRP-IR labeled fibers that crossed the border between the dermis and epidermis (crossing fibers) as well as the number of CGRP-IR fibers terminating in the epidermis using an Olympus BH microscope equipped with a Lucivid™ miniature monitor and Neurolucida™ software (MicroBrightField, Inc., Colchester, VT). Dermal and epidermal contours were drawn using the 2.5x objective and, using systematic scanning of the whole epidermal contour, the CGRP-IR fibers were identified using a 20x objective. Data from 6 rats were subsequently combined at 40 μm intervals and entered in Matlab (The Mathworks, Natick, MA) for the two-dimensional reconstruction of the foot sole. For the remaining rats, the number of crossing and terminal CGRP fibers from the 800 μm of the skin at the most medial and lateral side, starting at the transition point of the hairy to glabrous skin, and the center area (between footpads 3 and 4, see Figure 10) of the section were counted for statistical analysis of all the 30 sections. This enabled us to quantitate the number of CGRP fiber crossings and endings per mm^2^.

### Epidermal thickness

The effect of the SNI procedure on the epidermal thickness, defined as the vertical distance between the epidermal-dermal junction and the top of the outermost granular layer [49], was determined using an Olympus BH microscope equipped with a Lucivid miniature monitor and Neurolucida™ software (MicroBrightField, Inc., Colchester, VT). Five sections were randomly chosen from both the proximal and distal areas of the glabrous skin of a foot sole (total 10 sections). Definition of the lateral and medial side was the transition point of the hairy to the glabrous skin area and 375 μm from this point the epidermal thickness was measured. The epidermal thickness of the center area was determined at the center-most point of the transverse skin section but did not involve footpads.

### Statistical analysis

Per group, the results were averaged and compared with the average results of the other groups. Errors in the variations were assessed as the standard error of the mean (SEM). The unpaired *t*-test or one-way ANOVA with a Tukey post hoc test was performed for statistical comparison between groups, p < 0.05 was taken as significant.

## Competing interest

The authors have no conflict of interest that could compromise the conduct of this study or the reporting of the results.

## Authors' contributions

Conception and Design: D, H, W and R. Acquisition of data: D, K, H and F. Analysis: D, H and R. Interpretation of data: D, H, H, W and R. Drafting the manuscript: D, H, H, M, W and R. All authors read and approved the final manuscript.
